# Unmanned Aerial System-Based Weed Mapping in Sod Production Using a Convolutional Neural Network

**DOI:** 10.3389/fpls.2021.702626

**Published:** 2021-11-26

**Authors:** Jing Zhang, Jerome Maleski, David Jespersen, F. C. Waltz, Glen Rains, Brian Schwartz

**Affiliations:** ^1^Department of Crop and Soil Sciences, University of Georgia, Tifton, GA, United States; ^2^Department of Crop and Soil Sciences, University of Georgia, Griffin, GA, United States; ^3^Department of Entomology, University of Georgia, Tifton, GA, United States

**Keywords:** Bermudagrass, artificial intelligence, Fastai, ResNet, RGB imagery

## Abstract

Weeds are a persistent problem on sod farms, and herbicides to control different weed species are one of the largest chemical inputs. Recent advances in unmanned aerial systems (UAS) and artificial intelligence provide opportunities for weed mapping on sod farms. This study investigates the weed type composition and area through both ground and UAS-based weed surveys and trains a convolutional neural network (CNN) for identifying and mapping weeds in sod fields using UAS-based imagery and a high-level application programming interface (API) implementation (Fastai) of the PyTorch deep learning library. The performance of the CNN was overall similar to, and in some classes (broadleaf and spurge) better than, human eyes indicated by the metric recall. In general, the CNN detected broadleaf, grass weeds, spurge, sedge, and no weeds at a precision between 0.68 and 0.87, 0.57 and 0.82, 0.68 and 0.83, 0.66 and 0.90, and 0.80 and 0.88, respectively, when using UAS images at 0.57 cm–1.28 cm pixel^–1^ resolution. Recall ranges for the five classes were 0.78–0.93, 0.65–0.87, 0.82–0.93, 0.52–0.79, and 0.94–0.99. Additionally, this study demonstrates that a CNN can achieve precision and recall above 0.9 at detecting different types of weeds during turf establishment when the weeds are mature. The CNN is limited by the image resolution, and more than one model may be needed in practice to improve the overall performance of weed mapping.

## Introduction

Weeds are a persistent problem on sod farms. Herbicides to control different weed species are one of the largest chemical inputs (Satterthwaite et al., 2009; Wojciech and Landry, 2009; Yi, 2012) and often their control requires multiple applications throughout the growing season. A variety of annual and perennial broadleaf and grassy weeds are usually present in Georgia sod farms including annual bluegrass (*Poa annua*), goosegrass (*Eleusine indica*), crabgrass (*Digitaria* spp.), dallisgrass (*Paspalum dilatatum*), sedges (*Cyperus* spp.), spurge (*Euphorbia* spp.), chickweed (*Stellaria media* L.), and pigweed (*Amaranthus* spp.) (Colvin et al., 2013). Regulations limiting the broadcast application of certain chemicals in sod production (USEPA, 2009), due to concerns about the environmental impacts of the herbicide, create difficulty in effectively controlling weeds. Aside from the environmental cost of herbicides, there are significant financial costs in purchasing the herbicide and the labor and fuel used in application. Site-specific weed management, such as applying herbicides only where the weeds are located, instead of whole-field broadcast applications would significantly reduce herbicide use, thereby improving economic and environmental sustainability in sod production. The presence of weeds negatively affects turfgrass certification programs by increasing inspection times of sod that is being guaranteed as weed-free and uniform before being sold to consumers for uses such as sports fields, golf courses, and home lawns. Thus, the ability to quickly identify and respond to areas with weed issues is an attractive proposition for both sod growers and inspection agencies.

One of the key components for site-specific weed management is the generation of a weed map. Recent technical advances in unmanned aerial systems (UAS) have allowed for fast image acquisition and weed mapping using UAS in crops such as sunflower (*Helianthus* spp.) (Torres-Sánchez et al., 2013), cotton (de Castro et al., 2018), and rice (*Oryza sativa*) (Huang et al., 2018; Stroppiana et al., 2018). In these field crops, weed mapping was often conducted early in the growing season before canopy closure (Torres-Sánchez et al., 2013; López-Granados et al., 2016; Pérez-Ortiz et al., 2016; Stroppiana et al., 2018). Torres-Sánchez et al. (2013) evaluated image spatial and spectral properties for discriminating weeds in sunflower fields and reported adequate separation among weeds, crops, and bare soil using Excess Green Index, Normalized Green-Red Difference Index, and Normalized Difference Vegetation Index (NDVI) at a 30-m altitude. López-Granados et al. (2016) implemented object-based image analysis (OBIA) to extract the crop row and used both the relative position of vegetation to the crop row and spectral features to locate weeds. Successful late-season weed mapping using a UAS in oat fields was possible by taking advantage of greater spectral differences between oats and perennial weeds, as cereal crops become yellow during their senescence phase (Gašparović et al., 2020). Machine learning algorithms such as k-means clustering and random forest combined with OBIA were used for image classification.

However, only a few pieces of research have been conducted on how to best implement UAS-based weed mapping for sod production. Knowledge gained from the previous study of row crops is difficult to directly apply to turfgrass systems because they have unique challenges when it comes to weed mapping. First, there is no crop row in sod production to a pattern where the turfgrass should be. Second, regular mowing in sod production removes the morphological distinction of weeds, and it is not a common practice in other crops. Also, as a perennial crop, weeds are a year-round problem. Furthermore, and possibly most problematic, weed mapping in turfgrass production requires the differentiation of weeds against a green vegetation background instead of soil. Deep learning neural networks may be a good approach to address these challenges, and there is a growing set of literature developing weed image recognition models (Mahmudul Hasan et al., 2021). These often depend on high-resolution images of the weed leaf with or without background vegetation (Olsen et al., 2019; Espejo-Garcia et al., 2020; Hu et al., 2020). Yu et al. (2019a,b,c) reported several deep convolutional neural network (CNN) models that are exceptionally accurate (F1 score > 0.92, accuracy = 0.99) at detecting several broadleaf weeds in dormant and non-dormant Bermuda grass (*Cynodon* spp.) and perennial ryegrass (*Lolium perenne* L.) using images taken at the ground level (0.05 cm pixel^–1^). The best-performing image classifiers for detecting three broadleaf types in active-growing Bermuda grass including *Hydrocotyle* spp., *Hedyotis corymbosa*, and *Richardia scabra* were trained using the architecture VGGNet consisting of 16 layers (Yu et al., 2019b). VGGNet is a CNN architecture proposed in 2014 (Simonyan and Zisserman, 2014). These previous examples exploited either very high-resolution images or distinct cropping system features to aid in identifying weeds.

There is a lack of information to quantify the potential savings of using site-specific weed management in sod production, which will likely be critical before end-users such as farmers and certification agencies adopt this new technology. Thus, the objectives of this study were (1) to investigate weed-type composition and distribution through both ground and UAS-based weed surveys on sod farms and (2) to assess the feasibility of training and using a CNN for weed mapping in sod fields using UAS-based imagery. Therefore, our hypotheses were that (1) the percentage of the area without weeds was high even in a weed-infested area from human eyes and (2) a CNN can be trained with reasonable performance to detect the generic type of weed in the sod production field.

## Materials and Methods

### Ground Survey

**FIGURE 1:**
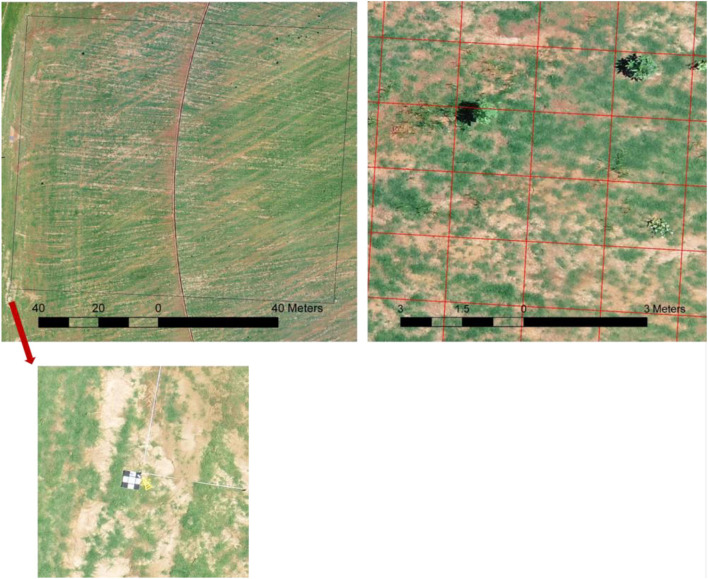
Example of the survey area conducted on Georgia sod farm in 2019. Top left: overlook of the survey with the size of 91 m by 91 m outlined by the black box and the ground target placed on the southwest corner (bottom); top right: one small section of the survey with the grid overlaid.

Turfgrass weed surveys were carried out on sod production fields, on six different occasions during the growing season in 2019 and 2020 (Table 1). Ground weed surveys were conducted shortly after UAS flights for ground truth labeling of the images for deep learning. For the ground survey, a grid was laid on the area where UAS flew over with sizes ranging from 30 to 91 m squares using measuring tapes. Four ground targets were placed on the four corners of the whole grid to help generate shapefile later during the image process and labeling (Figure 1). The cell size of each grid was 1.5 m by 1.5 m. People who conducted the survey walked through the area in one direction, visually assessed, and recorded every 1.5 m on a notepad whether or not a certain type of weed was present, and then the measuring tape was moved down 1.5 m in the other direction. Broad categories of weeds included broadleaf, grass weeds, and sedge. The category “grass weeds” present in our study included crabgrass, goosegrass, and dallisgrass. In one of the surveys, spotted spurge (*Euphorbia maculata*) was present, and it was separated as a different category due to its purple leaves and stems and unique appearance after close mowing in the sod fields.

**TABLE 1:** Summary of the training images from six surveys conducted on Georgia sod farms in 2019 and 2020.

Survey number	Turf species	Status of the field	Dominant weed types	Number of images
1	Bermuda grass	Establishing	Broadleaf, Sedge	3,570
2	Bermuda grass	Established	Broadleaf, Sedge, Crabgrass	1,600
3	Bermuda grass	Established	Broadleaf, Sedge, Crabgrass	1,600
4	Bermuda grass	Established	Broadleaf, Sedge	1,600
5	Bermuda grass	Establishing	Broadleaf, Goosegrass	530
6	Bermuda grass and Zoysia grass	Established	Broadleaf, Sedge, Spotted spurge	1,280

### Unmanned Aerial Systems Survey

Unmanned aerial system flights were conducted using DJI Phantom 4 Pro V2 (DJI, Shenzhen, China) equipped with a 20 megapixel red, green, and blue (RGB) camera. The image resolution was 4,864 × 3,648. The flights were conducted at 75% side and front overlap, and the flight altitudes ranged from 20 to 40 m, resulting in ground sampling distances of 0.57 cm–1.28 cm pixel^–1^. The flight times were between 10 a.m. and 4 p.m. with varying light conditions (clear, overcast, and partially cloudy). The flight plans were preprogrammed using DroneDeploy (DroneDeploy, Inc., San Francisco, CA, United States), which sets up the flight parameters such as path, altitude, and image overlap and sent out waypoints for autonomous flights.

### Image Process and Labeling

Raw images were processed through Pix4DMapper (Pix4D SA, Lausanne, Switzerland), and orthomosaics were generated using a standard workflow template – “Ag RGB.” The orthomosaic of each flight was further cropped into smaller images representing 1.5 m by 1.5 m cell size (Figure 1). Two main considerations were given to decide a proper cell size as follows: (1) 1.5 m by 1.5 m resulted in ∼200 pixels for each image which is needed to include important plant features and (2) the image size aligned with the ground survey cell size, which is practical for ground survey due to its intensiveness and time-consuming nature. The cropped images were labeled according to the ground survey results. Labels were divided into five classes including broadleaf, grass weeds, spotted spurge, sedge, and no weeds.

### Training a Convolutional Neural Network

Fastai framework was chosen to train and validate the multi-label image classifier. Fastai is built on PyTorch and provides a high-level application programming interface (API), which implements many of the best practices from literature, allowing data practitioners to quickly create and train deep learning networks to achieve state-of-the-art results (Howard and Gugger, 2020). A multi-label method was used instead of an object detection method for the following reasons: sections of the field were targeted for treatment rather than individual plants, and the multi-label image classifier is lighter weight, less data, and process-intensive, and easier to train and implement because the drawing of bounding boxes is not required as in the object detection method.

**FIGURE 2:**
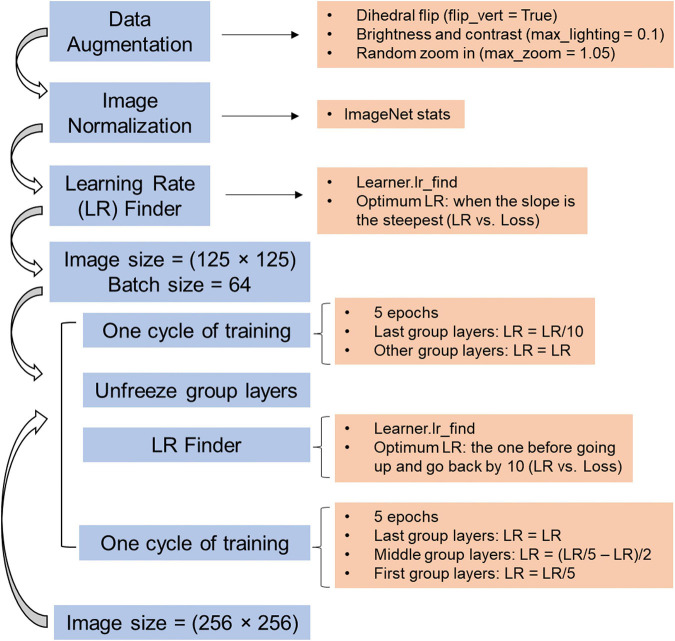
Schematic workflow for training image classifier in Fastai.

More than 10,000 images from 6 surveys (Table 1) were used to train a CNN. The training was conducted under a Windows 10 operating system, and the graphics processing unit (GPU) card was NVIDIA Quadro P4000. The images were divided into training (80%) and validation (20%) datasets. The architecture used was ResNet 34. Another architecture ResNet 50 was also tested and yielded a deeper CNN, but no improvement of the performance on the validation dataset was found (Supplementary Table 1). The general workflow is illustrated in Figure 2, including data augmentation, image normalization, finding the learning rate (LR), and cycles of training from lower image size to higher image size. Through the process, approximately 20 epochs were trained with variable LRs. LR was determined using an LR finder (Learner.lr_find), which launches an LR range test to help the practitioner select a good LR. The test trains the model with exponentially growing LR, stops in case of divergence and then plots the losses vs. LR with a log scale. A good LR is when the slope is the steepest (Howard, 2020). The change of loss for training and validation datasets during four phases of training is included in Supplementary Figure 1.

### Metrics, Thresholds, and Performance Comparison

The model output of the validation dataset is the number between zero and one indicating the confidence in the prediction for each class, the higher the number, the more probable the class. To assess precision vs. recall tradeoffs, a range of threshold values for accepting a positive result from the model between 0.2 and 0.5 was evaluated. The number of true positives (Tp), true negatives (Tn), false positives (Fp), and false negatives (Fn) were obtained. Metrics for accuracy, precision, and recall were further computed as follows (Sokolova and Lapalme, 2009):

Accuracy evaluates the average effectiveness of a classifier:


(1) $$\mathrm{Accuracy}\,\mathrm{per}\,\mathrm{class}=\frac{\mathrm{Tp}+\mathrm{Tn}}{\mathrm{Tp}+\mathrm{Fp}+\mathrm{Tn}+\mathrm{Fn}}$$

(2) $$\mathrm{Average}\,\mathrm{Accuracy}=\frac{\sum_{i=1}^{l}\mathrm{Accuracy}}{l}$$

Precision measures the number of correctly classified positive examples divided by the number of examples labeled by the system as positive:


(3) $$\mathrm{Precision}\,\mathrm{per}\,\mathrm{class}=\frac{\mathrm{Tp}}{\mathrm{Tp}+\mathrm{Fp}}$$

(4) $$\mathrm{Average}\,\mathrm{precision}=\frac{\sum_{i=1}^{l}\mathrm{Precision}}{l}$$

Recall measures the number of correctly classified positive examples divided by the number of positive examples in the data:


(5) $$\mathrm{Recall}\,\mathrm{per}\,\mathrm{class}=\frac{\mathrm{Tp}}{\mathrm{Tp}+\mathrm{Fn}}$$

(6) $$\mathrm{Average}\,\mathrm{recall}=\frac{\sum_{i=1}^{l}\mathrm{Recall}}{l}$$

In our use case, increasing precision will reduce the herbicide sprayed on non-weed areas, whereas increasing recall will ensure a more thorough control of the weeds (i.e., not missing any weeds). For sod growers using broadcast applications for weed control, emphasizing increased recall could enhance their confidence for early adoption of the technology. Thus, a metric Fbeta was used to evaluate the model by taking both the precision and recall into account using a single score (Sasaki, 2007):


(7) $$\mathrm{Fbeta}=\frac{\left(1+{\mathrm{beta}}^{2}\right)*\mathrm{Precision}*\mathrm{Recall}}{{\mathrm{beta}}^{2}*\mathrm{Precision}+\mathrm{Recall}}$$

(8) $$\mathrm{Average}\,\mathrm{Fbeta}=\frac{\sum_{i=1}^{l}\mathrm{Fbeta}}{l}$$

Beta = 2.0, referred to as the F2 score, is used to put more weight on recall than precision.

Metrics were computed separately for survey 1 and the other surveys. The field in survey 1 was under establishment, and the sod grower postponed herbicide application and mowing, resulting in relatively mature weeds. The larger weeds made for easier detection and better results. Metrics for survey 1 represent the case where weeds are relatively mature whereas the rest of the surveys represent more typical conditions with smaller weeds and more challenging conditions for weed detection. Recall was also used to compare system performance against human performance in order to identify opportunities to improve based on the existing dataset. Three human evaluators visually labeled the validation dataset for each class, and their recall was recorded.

## Results

### Ground Survey Results

**FIGURE 3:**
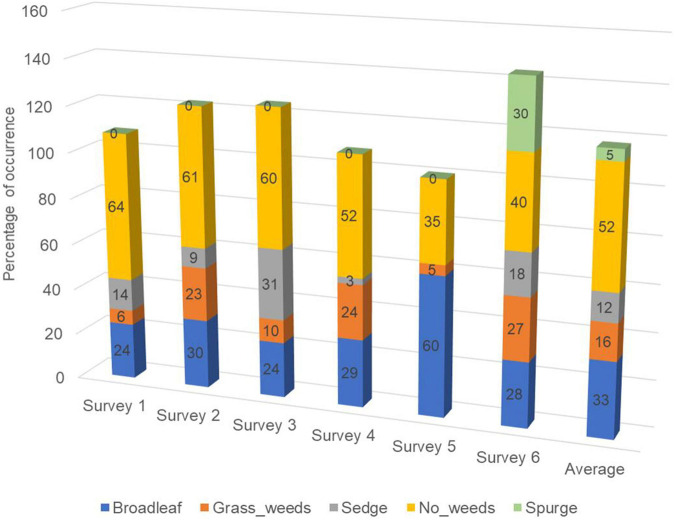
Percentage of area (cells) with different weed types presented in six surveys corresponding to the surveys summarized in Table 1. All surveys were conducted on Georgia sod farms in 2019 and 2020.

**FIGURE 4:**
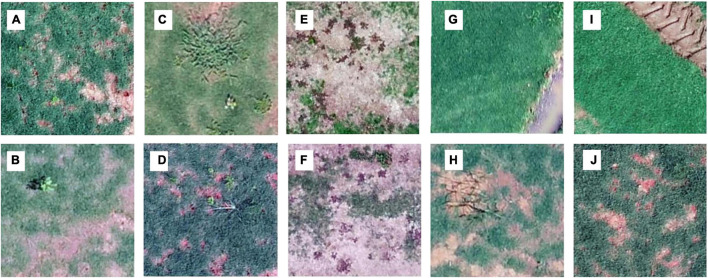
Examples of training images for each class: broadleaf **(A,B)**, grass weeds **(C,D)**, spotted spurge **(E,F)**, sedge **(G,H)**, and no weeds **(I,J)**. The images were obtained through different surveys on Georgian sod farms in 2019 and 2020.

A large portion (35–64%, 52% on average) of the 1.5 m by 1.5 m surveyed areas had no weeds present (Figure 3). Categories including broadleaf (Figures 4A,B), grass weed (Figures 4C,D), spotted spurge (Figures 4E,F), sedge (Figures 4G,H), and no weeds (Figures 4I,J) were recorded in the surveys. Areas of broadleaf and grass weeds accounted for 24–60% and 5%–27% of the total surveyed area, respectively. Sedge was only found in 3–31% of the total area. Spotted spurge was only found in survey 6 (30% of total area) where it could be detected by its purple leaves (Figures 4E,F).

### Performance of Image Classifier (Convolutional Neural Network) for Weed Mapping

Validation results of the CNN are listed in Tables 2, 3. Images from six surveys were collected under different sod growing stages including establishing, mature, and after harvest in order to train a more generalized model. When using a higher threshold value for the final decision, the precision of the CNN increased but its recall decreased. The CNN detected broadleaf, grass weeds, spurge, sedge, and no weeds at a precision of 0.68–0.87, 0.57–0.82, 0.68–0.83, 0.66–0.90, and 0.80–0.88, respectively, with varying threshold values from 0.5 to 0.2 (Table 2). Recall ranges for the five classes were 0.78–0.93, 0.65–0.87, 0.82–0.93, 0.52–0.79, and 0.94–0.99, respectively. Recall of detecting sedge was approximately 10–20% lower than when detecting other classes, indicating a higher number of false negatives. F2 scores were similar to recall due to its emphasis on the number of false negatives. Recall for sedge was elevated from 0.52 to 0.79 if the threshold value was set at 0.2, but the precision of detecting all classes decreased accordingly.

**TABLE 2:** Validation results of a multiple-class neural network trained on six surveys using architectures ResNet 34 for detection of weed types in sod production fields.

	Broadleaf	Grass weeds	Spurge	Sedge	No weeds	Avg.	Avg.T
*Threshold = 0.5*							
Precision	0.87	0.82	0.83	0.90	0.88	0.86	0.85
Recall	0.78	0.65	0.82	0.52	0.94	0.74	0.69
Accuracy	0.91	0.93	0.99	0.92	0.89	0.93	0.94
F2 score	0.80	0.68	0.82	0.57	0.93	0.76	0.72
*Threshold = 0.4*							
Precision	0.81	0.75	0.79	0.86	0.86	0.81	0.80
Recall	0.84	0.72	0.84	0.59	0.96	0.79	0.75
Accuracy	0.90	0.92	0.99	0.93	0.89	0.93	0.93
F2 score	0.84	0.72	0.83	0.63	0.94	0.80	0.76
*Threshold = 0.3*							
Precision	0.75	0.67	0.74	0.78	0.84	0.76	0.73
Recall	0.90	0.78	0.89	0.68	0.97	0.85	0.81
Accuracy	0.89	0.91	0.98	0.93	0.88	0.92	0.93
F2 score	0.86	0.76	0.86	0.70	0.94	0.83	0.80
*Threshold = 0.2*							
Precision	0.68	0.57	0.68	0.66	0.80	0.68	0.65
Recall	0.93	0.87	0.93	0.79	0.99	0.90	0.88
Accuracy	0.86	0.89	0.98	0.91	0.86	0.90	0.91
F2 score	0.87	0.78	0.87	0.76	0.94	0.85	0.82

*Ave.T is the average metric of the targeted classes including broadleaf, grass weeds, spurge, and sedge.*

**TABLE 3:** Validation results from survey 1 to the other 5 surveys of the multiple-class neural network trained using architectures ResNet 34 for detection of weed types in sod production fields.

	Broad-leaf	Grass weeds	Spurge	Sedge	No weeds	Broad-leaf	Grass weeds	Spurge	Sedge	No weeds
		
	**Survey 1 validation images**					**Surveys 2, 3, 4, 5, and 6 validation images**				
*Threshold = 0.5*										
Precision	0.93	0.96	na^*z*^	0.97	0.96	0.84	0.78	0.83	0.84	0.83
Recall	0.94	1.00	na	0.76	0.99	0.71	0.58	0.82	0.41	0.91
Accuracy	0.97	1.00	na	0.97	0.96	0.87	0.89	0.98	0.90	0.85
F2 score	0.94	0.99	na	0.79	0.98	0.74	0.61	0.82	0.46	0.89
*Threshold = 0.4*										
Precision	0.92	0.96	na	0.96	0.95	0.76	0.71	0.79	0.80	0.80
Recall	0.94	1.00	na	0.80	0.99	0.80	0.66	0.84	0.49	0.94
Accuracy	0.97	1.00	na	0.97	0.96	0.86	0.88	0.98	0.91	0.85
F2 score	0.94	0.99	na	0.83	0.98	0.79	0.67	0.83	0.53	0.91
*Threshold = 0.3*										
Precision	0.91	0.94	na	0.91	0.95	0.69	0.63	0.74	0.71	0.77
Recall	0.97	1.00	na	0.82	0.99	0.87	0.74	0.89	0.61	0.96
Accuracy	0.97	1.00	na	0.97	0.96	0.84	0.87	0.98	0.9	0.83
F2 score	0.96	0.99	na	0.84	0.98	0.82	0.72	0.86	0.63	0.91
*Threshold = 0.2*										
Precision	0.87	0.89	na	0.87	0.93	0.62	0.53	0.68	0.59	0.73
Recall	0.97	1.00	na	0.85	1.00	0.92	0.84	0.93	0.77	0.98
Accuracy	0.96	0.99	na	0.96	0.95	0.80	0.83	0.97	0.89	0.81
F2 score	0.95	0.98	na	0.85	0.98	0.84	0.75	0.87	0.72	0.92

*^z^na, not applicable. No spurge was present in survey 1.*

The CNN performed better in detecting validation images from survey 1 than from surveys 2–6 (Table 3). Precisions for detecting broadleaf, grass weeds, sedge, and no weeds in survey 1 were 0.87–0.93, 0.89–0.96, 0.87–0.97, and 0.93–0.96, respectively, with varying threshold values. Recall ranges for these four classes were 0.94–0.97, 1.00, 0.76–0.85, and 0.99–1.00, respectively. The metrics for validation images from surveys 2–6 were 10–40% lower in precision and 1–46% lower in recall than the metrics calculated from survey 1. It was noted that the CNN detected classes such as grass weeds and sedge in survey 1 at a much higher recall than in the other five surveys likely due to the larger and more mature weed size.

### Performance of Image Classifier Against Human Performance

**FIGURE 5:**
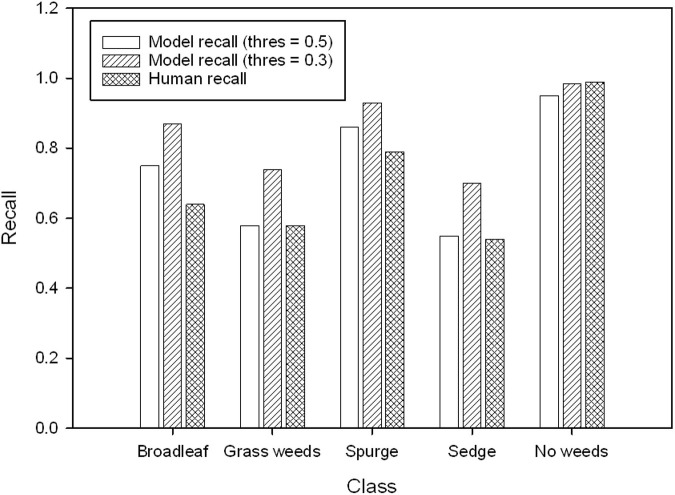
The comparison of recall (threshold values = 0.5 and 0.3) in validation result and recall from human performance (averaged from three evaluators).

**FIGURE 6:**
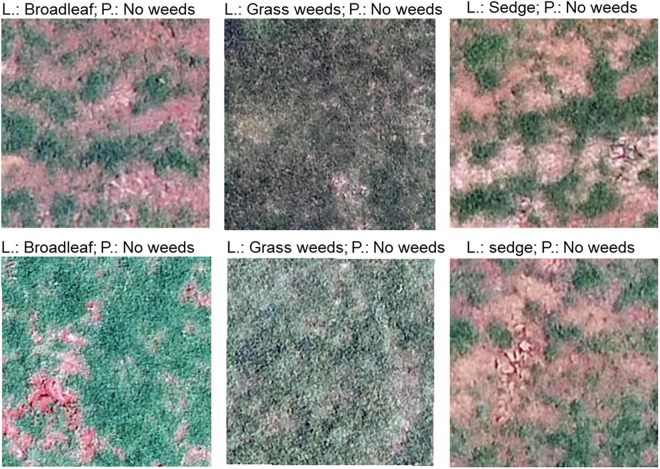
Examples of misclassified images by the convolutional neural network (CNN). L, true label; P, prediction.

The model performance indicated by recall was compared against human performance (Figure 5). The model recall was similar to human recall in detecting grass weeds, sedge, and no weeds when its threshold value was set at 0.5, but the model recall was higher in detecting broadleaf and spurge than human recall at this threshold. The model was able to detect more weed targets than human eyes if the threshold value was set at 0.3. The lowest human recall was for detecting sedges at 0.54, indicating approximately that half of the sedge targets were not visually identifiable by human eyes. Some examples of images labeled in class broadleaf, grass weeds, and sedge, but not visible to human eyes are demonstrated in Figure 6.

## Discussion

To our knowledge, this is the first study investigating the use of UAS-based images and a deep learning model for weed mapping on sod farms. According to the ground survey result, on average, 52% of each field had no weeds present, which demonstrates the potential for reducing postemergence herbicide use if site-specific weed management can be properly adopted. These reductions can be economically and environmentally impactful. The advantages of using UAS with a simple RGB camera are manifold. Once the detection model and relevant software are available, UAS can cover large fields and generate weed maps in a relatively short time. By integrating this technology into a weed management program, sod growers will have the capability to quickly document problematic areas in the field and make sound treatment decisions. Typically, postemergence herbicides, such as 2,4-D, carfentrazone, dicamba, and simazine, are uniformly sprayed across Bermuda grass fields to provide control of various broadleaf weeds (McCalla et al., 2004; Yu et al., 2019b). By moving broadcast applications to targeted applications, growers will be more competitive with lower herbicide costs. Furthermore, this technology will reduce their environmental footprint by minimizing the pesticides used on sod farms, helping improve the sustainability of the industry.

The CNN trained using ResNet 34 demonstrated the capability to extract color, texture, and shape features (Deng et al., 2010; Grinblat et al., 2016) of different classes of weeds, achieving precision and recall of above 0.9 with the exception of sedges in an establishing field or the larger and mature weeds found in survey 1. The dominant broadleaf weed in survey 1 was pigweed (*Amaranthus* spp.) in varying sizes and growth stages. Precision for detecting broadleaf was 0.93 when the threshold *p*-value was set at 0.5, indicating that only 7% of the targets were misclassified. Results on recall exhibited that approximately 3–6% broadleaf targets were not detected. Yu et al. (2019a) reported a VGGNet model which detected three broadleaf types in Bermuda grass with precision ranging from 0.91 to 0.97 and recall ranging from 0.97 to 1.00. Their model detected almost all the targets, possibly due to the extremely high-resolution images (0.05 cm pixel^–1^) used to train their model. The CNN in our study only yielded comparable results in survey 1, likely because the weeds were more mature, offsetting the 10–20 times lower (0.57 cm–1.28 cm pixel^–1^) ground sampling distance than reported by Yu et al. (2019a). Metrics for detecting sedges indicate that 97% of the identified targets (precision) were accurate, and approximately 24% of the sedge targets in the surveyed area were not identified. Sedges are more challenging to detect than broadleaf due to their grass-like morphology: narrow leaf blades and broad ranges of types including nutsedges, annual and perennial sedges, and kyllinga (McCullough et al., 2015). Broadcast postemergence herbicides such as flazasulfuron, halosulfuron, imazaquin, sulfentrazone, sulfosulfuron, and trifloxysulfuron-sodium may still be needed to control sedges given the limitations of our CNN at this time (McElroy and Martins, 2013; McCullough et al., 2015).

Six surveys in our study were conducted in multiple sod fields with different surface conditions (establishing and mature fields) and weed types. It is not surprising that the practical effectiveness of the CNN was lower in the validation dataset of surveys 2–6 than for that of the first survey. Over the whole validation dataset, the CNN detected 78% of broadleaf, 65% grass weeds, 82% spurge, 52% sedge, and 94% no weeds when the threshold *p*-value was set at 0.5. The recall from human evaluators was generally lower than model recall, indicating that the limiting factor was the image resolution and a number of the smaller weeds were simply not visible in these cases. This also explained why a deeper architecture such as ResNet 50 did not improve the model performance. Nevertheless, by lowering the threshold to 0.3, 10% more targets can be identified at the expense of reduced precision. This might be a good option in practice, allowing the growers to balance the cost vs. control. During the surveys in this research, it was noted that weeds were either sporadically distributed across the field or followed a linear pattern, possibly resulting from spread from tractor tires or mowers. Another explanation could be skipped in previous preemergence and postemergence herbicide applications. In this study, the identification of seemingly randomly distributed weeds in a sod production field using the weed map generated by the CNN would be of great economic and environmental benefit. Diverse datasets are needed to train generalized models that perform well in different scenarios because the dynamics of weed pressure are fluid and ever-changing. Given that this study was one of the first attempts to generate a weed map using deep learning in turfgrass, there is less information to compare to at a similar scale and resolution.

The comparison between model recall and human recall suggested that the model performance is limited by image resolution. Higher image resolution would improve the performance of the model but requires greater computing power and either expensive cameras or lower and longer flight time. In some cases, it is a challenge to conduct low-altitude flights due to the close proximity of power lines or trees. Our results indicate that it is difficult to incorporate UAS-based weed mapping at a very early stage of weed treatment when the weeds are still immature or relatively small in size. In the future, technology continues to improve, and UAS-based weed mapping would be improved by higher resolution cameras or fully automated drone fleets flying close to the ground. In addition, a ground-based camera system on tractors or center pivot irrigation system could have much higher resolution and would be ideal for weed mapping if the images were collected in a consistent, timely, and automatic manner.

It remains uncertain whether a single model is sufficient to cover the whole spectrum of weed scenarios in sod production due to the complexity of the turfgrass-weed interactions during the entire growing season. During winter dormancy, however, a separate CNN will be needed to map winter weeds including *Poa annua* and ryegrass (*Lolium* spp.) along with some broadleaf weeds in production fields. Dormant turfgrass provides a brown background with more contrast for weed detection. Even after the CNN for weed mapping is available, there are several hurdles before the implementation of site-specific herbicide applications become routine, including the development of software to generate weed maps using the CNN, ensuring the location accuracy of the position of target weed, and the integration of the weed map into multiple sprayer systems.

## Summary

This study included the survey of several sod production fields for broadleaf, grass weeds, spurge, and sedge weed-type composition and areas of infestation, both from the ground level and using UAS, demonstrating the potential of herbicide savings if site-specific weed management is properly adopted. This study successfully trained a CNN for weed mapping using UAS-based imagery and high-level API implementation of a deep learning library. The performance of the CNN was overall similar to, and in some classes (broadleaf and spurge) better than, human identification as indicated by the metric recall. In general, the CNN detected different types of weeds at precision ranging from 0.57 to 0.90 and recall from 0.52 to 0.99 when using UAS images with similar resolution in this study (0.57 cm–1.28 cm pixel^–1^). Furthermore, it was demonstrated that the CNN can achieve precision and recall above 0.9 for detecting different types of weeds under establishing field conditions when they are larger and more mature. Image resolution is currently the major limiting factor to further improvement of the CNN, with one possible solution being ground-level scouting. Due to the complex ecology and biology of the weeds typically found on sod farms, different models may be needed in practice to improve the overall performance of weed mapping and the eventual targeted, site-specific application of herbicides in these production systems.

## Data Availability Statement

The original contributions presented in the study are included in the article/Supplementary Material, further inquiries can be directed to the corresponding author.

## Author Contributions

JZ designed the study, conducted the survey, analyzed the images, and wrote the manuscript. JM conducted the survey, provided the critical technical support on model training, and wrote the manuscript. BS, GR, DJ, and FW provided constructive suggestions on the study design and manuscript. BS and DJ helped to conduct the survey and provided support in coordination activities. All authors contributed to the article and approved the submitted version.

## Conflict of Interest

The authors declare that the research was conducted in the absence of any commercial or financial relationships that could be construed as a potential conflict of interest.

## Publisher’s Note

All claims expressed in this article are solely those of the authors and do not necessarily represent those of their affiliated organizations, or those of the publisher, the editors and the reviewers. Any product that may be evaluated in this article, or claim that may be made by its manufacturer, is not guaranteed or endorsed by the publisher.
