# Supplementary material for: Unmanned Aerial System-Based Weed Mapping in Sod Production Using a Convolutional Neural Network
Source: Front Plant Sci. 2021 Nov 26;12:702626. doi: 10.3389/fpls.2021.702626 (PMC8660967; doi:10.3389/fpls.2021.702626)
Supplement: Supplementary file 1 [file Data_Sheet_1.docx]

| Supplementary Table 1. Validation results of multiple-class neural network trained on six surveys using architectures resnet-50 for detection of weed types in sod production fields. | | | | | | | | |
| --- | --- | --- | --- | --- | --- | --- | --- | --- |
|  | Broadleaf | Grass | Spurge | Sedge | No | Avg. | Avg.T | |
|  | | Weeds |  |  | Weeds |  |  |  |
| *Threshold = 0.5* | |  | | | | | |  |
| Precision | 0.87 | 0.84 | 0.77 | 0.88 | 0.86 | 0.84 | 0.84 |  |
| Recall | 0.75 | 0.58 | 0.86 | 0.55 | 0.95 | 0.74 | 0.68 |  |
| Accuracy | 0.90 | 0.92 | 0.99 | 0.93 | 0.89 | 0.92 | 0.93 |  |
| F2 score | 0.81 | 0.69 | 0.81 | 0.67 | 0.91 | 0.78 | 0.75 |  |
| *Threshold = 0.4* | |  |  |  |  |  |  |  |
| Precision | 0.82 | 0.80 | 0.76 | 0.82 | 0.83 | 0.81 | 0.80 |  |
| Recall | 0.82 | 0.69 | 0.88 | 0.62 | 0.97 | 0.80 | 0.75 |  |
| Accuracy | 0.90 | 0.93 | 0.99 | 0.93 | 0.87 | 0.92 | 0.94 |  |
| F2 score | 0.82 | 0.74 | 0.82 | 0.71 | 0.90 | 0.80 | 0.77 |  |
| *Threshold = 0.3* | |  |  |  |  |  |  |  |
| Precision | 0.75 | 0.71 | 0.75 | 0.75 | 0.82 | 0.76 | 0.74 |  |
| Recall | 0.87 | 0.74 | 0.93 | 0.70 | 0.99 | 0.85 | 0.81 |  |
| Accuracy | 0.88 | 0.92 | 0.99 | 0.93 | 0.87 | 0.92 | 0.93 |  |
| F2 score | 0.80 | 0.73 | 0.83 | 0.73 | 0.89 | 0.80 | 0.77 |  |
| *Threshold = 0.2* | |  |  |  |  |  |  |  |
| Precision | 0.68 | 0.64 | 0.72 | 0.68 | 0.79 | 0.70 | 0.68 |  |
| Recall | 0.91 | 0.82 | 0.93 | 0.80 | 0.99 | 0.89 | 0.87 |  |
| Accuracy | 0.86 | 0.90 | 0.98 | 0.92 | 0.85 | 0.90 | 0.92 |  |
| F2 score | 0.78 | 0.72 | 0.82 | 0.74 | 0.88 | 0.79 | 0.76 |  |
| Ave.T is the average metric of the targeted classes including broadleaf, grass weeds, spurge, and sedge. | | | | | | | | |


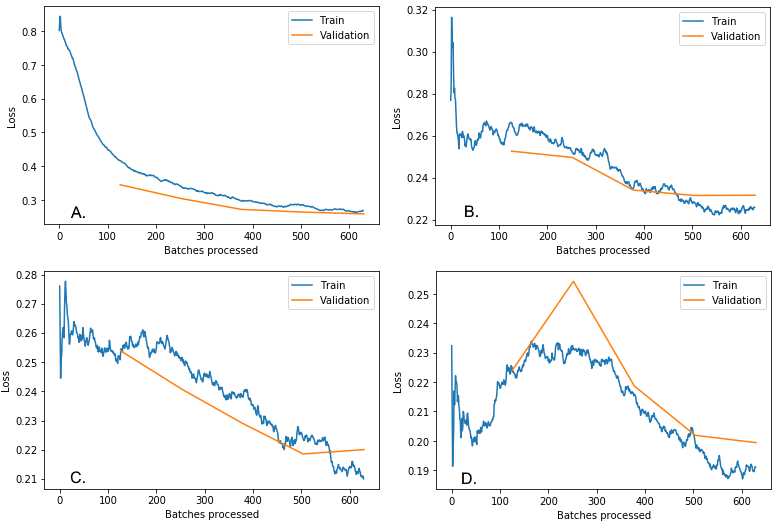


Supplementary Figure 1. Loss in training and validation data sets during four phases of training: A) first cycle of training on image size 125 × 125; B) second cycle of training on image size 125 × 125; C) first cycle of training on image size: 256 × 256; and D) second cycle of training on image size 256 × 256. Loss function used was BCEWithLogitsLoss (source: https://github.com/fastai/fastai/blob/master/fastai/losses.py#L84).
